# Comorbidities in Chronic Obstructive Pulmonary Disease from Assessment to Treatment

**DOI:** 10.1155/2014/414928

**Published:** 2014-04-02

**Authors:** Enrico M. Clini, Piera Boschetto, Mitja Lainscak, Wim Janssens

**Affiliations:** ^1^Department of Medical and Surgical Sciences, University of Modena Reggio Emilia, 41100 Modena, Italy; ^2^Department of Medical Sciences, University of Ferrara, 44100 Ferrara, Italy; ^3^University Clinic of Respiratory and Allergic Diseases, 4204 Golnik, Slovenia; ^4^Respiratory Division University Hospital, KU Leuven, Herestraat 49 3000 Leuven, Belgium

In recent years several studies in the field of chronic obstructive pulmonary disease (COPD) have addressed the impact of comorbidities on the phenotypic presentation of an individual patient [1]. Epidemiological studies have investigated the prevalence of comorbidities in COPD patients and have reported clear associations between COPD and other chronic diseases, such as cardiovascular, cancer, and metabolic diseases as the most frequent ones. Since then the awareness for coexisting diseases in COPD patients has been steadily growing and their role on increased morbidity, worse prognosis, and higher economic burden has currently been recognized.

Notwithstanding, the clinical and pathophysiological links between COPD and other chronic conditions remain controversial and are largely unknown. Some authors suggest that COPD may favor the development of other diseases via the spilling of inflammatory mediators from the lung to the systemic circulation. They indirectly support the idea of a causal relationship between COPD and its comorbid conditions. Others claim that the association of COPD and coexisting diseases is only based on the presence of common risk factors [2], and there is also cumulating evidence about activation of neurohumoral response [3]. In particular, metabolic abnormalities and physical inactivity on top of smoking may accumulate and intensify as COPD and its coexisting diseases progress. From this point of view, the term “multimorbidity” better reflects the concept of chronic complex patients with COPD rather than that of COPD patients with chronic comorbidities [4]. Moreover, advantageous to the multimorbidity concept is that its recognition in elderly adults also highlights the role of risk factors and premorbid conditions in COPD as unexplored pathways for intervention [5]. A comprehensive and individualized approach is therefore warranted, and rehabilitation in its broadest meaning may appear as best interventional process to assess, care, and manage according to this holistic view [6, 7].

The articles published within the special issue tackle these concepts and review some important areas of both assessing the presence of relevant coexisting conditions in COPD, as well as evaluating the effect of comprehensive care dedicated to these patients. In particular, the analysis of specific phenotypic clusters of these individuals, the presence of specific conditions which may worsen prognosis and quality of life, such as musculoskeletal disorders and dysfunction, the cognitive impairment, and the cardiovascular problems are reported. Moreover, the effectiveness of a rehabilitation course and associated problems, in such complex patients, is also reported in separate contributions.

In conclusion, actual evidence suggests that patients with multimorbidity represent a huge part of the elderly population, being COPD one component, not necessarily the most important, of this clinical phenotype that requires a comprehensive assessment, care, and long-term management. The respiratory community should recognize this complexity of chronic care in patients with COPD, and health care providers should carefully consider all strategies of primary and secondary prevention in the adult population.

*Enrico Clini* *Piera Boschetto* *Mitja Lainscak* *Wim Janssens*
